# Obesity and clinical severity in patients with COVID-19: a scoping review protocol

**DOI:** 10.1186/s13643-021-01603-x

**Published:** 2021-02-07

**Authors:** Marcela Larissa Costa, Carlos Adriano Santos Souza, Ana Caroline Cardoso Silva, Dayane Franciely Conceição Santos, Edilene Fernandes Nonato, Francismayne Batista Santana, Giselle dos Santos Dias, Jessyca Teles Barreto, Laís Santos Costa, Maria Carolina Barros Costa, Tamila das Neves Ferreira, Jeniffer Santos Santana, Raquel Simões Mendes-Netto, Tereza Virgínia Silva Bezerra do Nascimento, Marco Antônio Prado Nunes, Márcia Ferreira Cândido de Souza

**Affiliations:** 1 Nutrition Department of Federal University of Sergipe, Marechal Rondon Avenue, Rosa Elze, São Cristóvão, Sergipe 49100-000 Brazil; 2 University Center AGES – UniAGES, University Avenue, Number 23, Park of the Palms, Paripiranga, Bahia 48430-000 Brazil; 3 Tiradentes University, Murilo Dantas Avenue, Number 300, Farolândia, Aracaju, 49032-490 Brazil; 4 University Hospital of Sergipe, Federal University of Sergipe, Cláudio Batista Street, Cidade Nova, Aracaju, Sergipe 49060-108 Brazil; 5 Medical Department of University Hospital of Sergipe, Federal University of Sergipe, Cláudio Batista Street, Cidade Nova, Aracaju, Sergipe 49060-108 Brazil; 6 Nutrition Department of University Hospital of Sergipe, Federal University of Sergipe, Cláudio Batista Street, Cidade Nova, Aracaju, Sergipe 49060-108 Brazil

**Keywords:** Obesity, COVID-19, Risk factor, Severity

## Abstract

**Background:**

Coronavirus disease (COVID-19), caused by the severe acute respiratory syndrome coronavirus 2 strain, was first identified in late 2019 in China. The outcomes of patients affected by the virus can worsen, developing acute respiratory failure and other serious complications, especially in older individuals and people with obesity and comorbidities. Thus, obese patients tend to have a more severe course of COVID-19. Thus, this review aims to synthesize the evidence in the literature that associates COVID-19 and the severity of clinical outcomes in infected obese patients.

**Methods:**

This protocol was designed following the Preferred Reporting Items for Systematic Reviews and Meta-Analyses Protocols Statement. Scientific and gray literature will be systematically selected from PubMed/MEDLINE, Latin American Literature in Health Sciences, Online Scientific Electronic Library, Scopus, ScienceDirect, Web of Science, Embase, and Cochrane. The selection of articles will be limited to studies published in English, Portuguese, and Spanish from December 2019 onwards. The main clinical outcomes will be clinical severity in obese patients with COVID-19 as tachypnea (respiratory rate, ≥ 30 breaths per minute), hypoxemia (oxygen saturation, ≤ 93%), the ratio of the partial pressure of arterial oxygen to fraction of inspired oxygen (< 300), lung infiltrate (> 50% of the lung field involved within 24–48 h), diagnosis of the severe acute respiratory syndrome, need of invasive mechanical ventilation, and mortality. Two reviewers will independently screen all citations, full-text articles, and abstract data. Selection bias will be minimized by excluding studies published before December 2019. Conflicts will be resolved through a third reviewer and consensus-building. Moreover, findings will be reported using narrative synthesis and tabulation of the summaries.

**Discussion:**

Given the need for early detection of the possible implications and treatment for patients with obesity diagnosed with COVID-19, the scoping review will be useful to capture the state of the current literature, identify the gaps, and make recommendations for future research for directing the conduct and optimization of therapies in these patients by the multiprofessional teams.

**Systematic review registration:**

Open Science Framework: https://osf.io/xrkec

**Supplementary Information:**

The online version contains supplementary material available at 10.1186/s13643-021-01603-x.

## Background

Coronavirus disease 2019 (COVID-19), caused by the severe acute respiratory syndrome coronavirus 2 (SARS-CoV-2) strain, was first identified in late 2019 in Wuhan, China, in a group of patients with pneumonia due to an unknown cause [1]. A greater number of countries were affected in 2020, with increasing growth in the epidemiological curve of the disease. Consequently, COVID-19 has been officially declared a pandemic by the World Health Organization on March 11, 2020 [2].

The clinical manifestations of COVID-19 can be classified as asymptomatic and symptomatic, generally causing mild symptoms (e.g., fever, cough, sputum production, shortness of breath, and headache). However, patients affected by the virus can worsen, developing respiratory failure and other serious complications, especially in older individuals and people with obesity and comorbidities [3–5].

Obesity is often associated with noncommunicable diseases and conditions such as systemic arterial hypertension and cardiovascular disease [6]. Obese individuals have an increased risk of hospitalization, severe illness, and mortality, possibly associated with cardiovascular comorbidities, chronic inflammatory status, and immune response to infection [7]. The immune system is largely related to the inflammation caused by adipose tissue, resulting from obesity, and, therefore, its response is often impaired when associated with obesity [8].

The contribution mechanism of obesity to COVID-19 complications is not yet defined. However, several causes can influence the problem because obese patients present harmful respiratory physiology, including the reduction of the functional residual capacity, as well as hypoxemia and ventilation and perfusion abnormalities [9].

Some studies show that obese people infected with COVID-19 have a high frequency of admission to the intensive care unit and the need of using invasive mechanical ventilation in addition to the association between the highest mortality rates and body mass ratio [10, 11]. Thus, obese patients tend to have a more severe course of COVID-19. Thus, this review aims to synthesize how the literature associates COVID-19 with the severity of the clinical outcomes in infected obese patients.

## Methods

The six-stage Arksey and O’Malley framework [12] and Levac et al. [13] will be considered to performing this scoping review. Moreover, it is being reported following the recommendations of the Preferred Reporting Items for Systematic Reviews and Meta-Analyses (PRISMA) Protocols statement [14] (see checklist in Supplementary file 1). This review will be completed following the methods outlined by the Joanna Briggs Institute’s Method Manual for Scoping Review [15] and the PRISMA Extension for Scoping Reviews (PRISMA-ScR) [16]. These types of reviews are useful for examining emerging evidence when not yet clear and other more specific issues that can be reliably posed and addressed [15]. This protocol was reviewed by the members of the research team and has been registered on the Open Science Framework (registration link: https://osf.io/xrkec).

### Framework

The framework, proposed by Arksey and O’Malley [12] and further developed by Levac et al. [13], details six different stages in the process of conducting a scoping review: (1) identifying the research question, (2) identifying relevant studies, (3) selecting the studies, (4) charting the data, (5) reporting the results, and (6) expert consultation. Stage 6 is considered optional by the authors [12] and will not be carried out in this scoping review. We will also include the quality appraisal step to assess the quality of the included studies as recommended by Levac et al. [13].

#### Framework stage 1: identifying the research question

The research questions are as follows:

1. What is known from existing literature about the clinical severity in obese patients diagnosed with COVID-19?
2. What are the results related to serious or fatal complications in obese patients with COVID-19?
3. What are the main comorbidities related to obese patients who presented complications related to COVID-19?
4. How does obesity influence the worsening of respiratory parameters and the need for mechanical ventilation in patients diagnosed with COVID-19?

#### Framework stage 2: identifying relevant studies

Available studies in the scientific literature between December 2019 and onwards will be identified from PubMed/MEDLINE, Latin American Literature in Health Sciences, Online Scientific Electronic Library, Scopus, ScienceDirect, Web of Science, Embase, and Cochrane. For identification, research was conducted with the MeSH and DeCS terms with the following descriptors: (“covid-19” OR “covid-19 virus infection” OR “SARS-CoV-2 infection” OR “COVID-19 virus disease” OR “severe respiratory acute” OR “syndrome coronavirus 2” OR “severe acute respiratory syndrome coronavirus 2” OR “2019-nCoV” OR “SARS-CoV-2” OR coronavirus) and (mortality or hospital mortality or hospital) and comorbid* and obes*. The snowball sampling method will be used for references. This sampling method involves primary data sources, naming other potential primary data sources to be used in the research [17].

##### Eligibility criteria

Any evidence that met the Population, Intervention, Comparison, Outcomes, Context, and Study design (PICOCS) [18] criteria will be included in this review:

Population:

- Obese patients (body mass index ≥ 30 kg/m^2^), adults and elderly, infected with COVID-19, and with or without comorbidities.

Intervention:

- No restriction on interventions.

Comparison:

- Patients with normal-weight. For articles that have a descriptive observational design, no comparison is necessary.

Outcomes:

- Articles that report the severity of clinical outcomes in obese patients, with or without comorbidity, and related to SARS-CoV-2 infection [19], such as tachypnea (respiratory rate, ≥ 30 breaths per minute), hypoxemia (oxygen saturation, ≤ 93%), the ratio of the partial pressure of arterial oxygen to fraction of inspired oxygen (< 300), and lung infiltrate (> 50% of the lung field involved within 24–48 h). Moreover, other clinical outcomes will be obesity, severe acute respiratory syndrome diagnosis, and the need for invasive mechanical ventilation and mortality.

Context:

- Articles published in English, Portuguese, and Spanish will be included. Studies available in the literature will be identified between December 2019 and onwards.

Study design:

- This review will consider the designs of experimental studies, including randomized clinical trials, nonrandomized clinical trials, expert opinion, cohort study, and cross-sectional studies. In addition, analytical observational studies will be considered, including prospective and retrospective cohort studies, case-control studies, and cross-sectional analytical studies. This research will also consider descriptive observational study projects, including case series, individual case reports, and descriptive cross-sectional studies, for inclusion. Furthermore, editorials, opinion articles, reviews, systematic reviews, and gray literature will be considered for inclusion.

##### Exclusion criteria

Articles or studies will be excluded if full text cannot be obtained or if the language used was not in English, Portuguese, and Spanish.

#### Framework stage 3: study selection

The titles, abstracts, and articles in full will be evaluated by two independent reviewers. Three categories (*yes*, *no*, and *maybe*) were used in the selection of the title, abstract, and full text. The study will be selected for full-text evaluation in case of doubts (category *maybe*). At this stage, the divergences will be resolved by consensus between the two reviewers. Moreover, two reviewers will work independently to extract data, and the extracted data will be charted into a Microsoft Excel form as shown in Supplementary file 2. The review team will have training on how to use Microsoft Excel before the study begins to ensure the calibration of collection methods. The full search of the data will proceed only with *x* > 75% agreement across the team. If the values found were below the agreed value, the doubts will be clarified and the training exercise will be repeated. Consequently, conflicts will be resolved by a third reviewer.

A pilot of 20 studies will be carried out in this stage to calibrate the collection method and the agreement level. The research will be in accordance with the recommendations of the Peer Review of Search Strategies 2015 guidelines [20] according to the following variables: translation of the research question, Boolean operators and proximity, subject titles (database-specific), word text search, and limits and filters.

Selection bias will be minimized by excluding studies published before December 2019. However, the cutoff period was selected because the new coronavirus was identified in late 2019.

#### Framework stage 4: charting the data

The reviewers are experts in electronic research, systematic review, and scoping review. The data extracted will be discussed by the research team then summarized and tabulated in themes that addressed the research questions. Selection divergences will be resolved through a third reviewer and consensus-building. Moreover, Cohen’s kappa statistics will be used to measure reliability among evaluators. The information to be extracted is available in Supplementary file 2.

The reviewers will pilot the model on a sample of the included studies (10% of the selected studies) to ensure that the data extraction form is applied consistently. If necessary, the form will be revised and adjusted according to the course of the review process. The reviewers will extract and plot the data for each article included independently (two reviewers per article) based on the data extraction model.

#### Framework stage 5: collating, summarizing, and reporting the results

The study selection process will be summarized in a flowchart adapted from the PRISMA statement [14]. The data from each paper (e.g., study characteristics, participants, outcomes, findings, and limitations) will be used to build evidence tables of an overall description of the included studies.

The evaluation and characterization of the results will make it possible to relate obesity and its characteristics as an exponentially important factor in determining the risk of severity and the associated clinical parameters in patients with SARS-CoV-2. The data from the data collection process will be quantitatively summarized using a simple numerical count and qualitatively drawn on the descriptive-analytical method using thematic analysis and visual representations (including maps or diagrams). The quantitative results will be presented in a table format, followed by a narrative section containing the main theories collected from observational studies, intervention, and expert opinion papers published on the possible causes of the clinical severity in obese patients diagnosed with COVID-19.

Regarding the expert opinion articles, an evaluation of the information available will be extracted after a group discussion with the authors. The available literature will be carried out to explain the phenomenon studied. Moreover, a narrative account of the findings will be presented, and the thematic content analysis will be employed to extract the themes, which will be critically examined concerning the study research question, the aim of the study, literature, and gaps for future research (see the data extraction of editorials and expert opinion articles in Supplementary file 2). This will help to identify all relevant findings and themes to address the research question.

### Quality and critical appraisal

The quality of the articles will be assessed using the National Heart, Lung, and Blood Institute instruments for controlled intervention studies, systematic reviews and meta-analyses, observational cohort and cross-sectional studies, case-control studies, and case series studies [21] (Supplementary file 3). Regardless of quality score, papers will be included in the final review and analyses but descriptively summarized according to the risk of bias. Corresponding authors will be contacted if insufficient details exist to confidently assess the risk of bias in individual studies.

For the expert opinion articles, the critical appraisal will be independently conducted by two members of the research team to identify potential bias, strengths, and limitations of included studies.

### Statistical analysis

The Statistical Package for the Social Sciences, version 17.0 will be used to calculate the kappa index to verify agreement in the selection of studies included among the authors, reducing the chance of a study loss and the possibility of bias.

### Dissemination

This project is part of a research program on COVID-19 and obesity. The results will serve as a basis for the development of treatment strategies for obese patients diagnosed with COVID-19. The idea of this review came through the Nutrition and Obesity research group at the University Hospital of the Federal University of Sergipe. The results of the review will be shared through peer-reviewed publications, presentations, workshops, and educational institutions.

## Discussion

The scoping review seeks to synthesize the research evidence by mapping the existing literature in a field of interest in terms of volume, nature, and characteristics of primary research. Furthermore, they are usually carried out to examine the extent, scope, and nature of the activity. This type of research shares several processes like systematic reviews because they use rigorous and transparent methods to comprehensively identify and analyze all literature related to a research question [14, 15].

According to the Centers for Disease Control and Prevention, obesity is associated with impaired immune function, disease severity, and the risk of mortality from COVID-19 [22]. The underlying metabolic and inflammatory factors of individuals with obesity also play a considerable role in the manifestation of serious lung diseases. Susceptibility to acute respiratory distress syndrome, the leading cause of COVID-19 mortality, is significantly greater among obese individuals [23].

This research will synthesize evidence from the available studies related to obesity and COVID-19 severity. Therefore, given the need for early detection of possible implications and treatment for patients with obesity, the evidence will be useful for the conduction of direction and optimization of therapies in these patients by multiprofessional teams, given the high frequency of intensive care and high risk of mortality in these patients, to improve therapeutic interventions and reduce unfavorable outcomes.

## Limitations

The search for studies will be performed in certain databases, which may restrict the inclusion of articles that are not in the selected databases. In addition, a language limitation exists because the search for manuscripts will be done in three languages (Portuguese, English, and Spanish), which restricts selection and findings.

## Supplementary Information

**Additional file 1:.** PRISMA-P 2015 Checklist

**Additional file 2:.** Data extraction form

**Additional file 3:.** Study quality assessment tools

## Data Availability

Not applicable

## Abbreviations

ARDS: Acute respiratory distress syndrome
BMI: Body mass index
COVID-19: Coronavirus disease
DeCS: Health Sciences Descriptors
ICU: Intensive care unit
IMV: Invasive mechanical ventilation
JBI: Joanna Briggs Institute
LILACS: Latin American Literature on Health Sciences
MESH: Medical Subject Headings
NHLBI: National Heart Lung and Blood Institute
PRESS: Peer-review of search strategies
PICOCS: Population, Intervention, Comparison, Results, Context, and Study design
PRISMA: Preferred Reporting Items for Systematic Review and Meta-Analysis
PRISMA-P: Preferred Reporting Items for Systematic Review and Meta-Analysis Protocols
PRISMA-ScR: Preferred Reporting Items for Systematic Review and Meta-Analysis Extension for Scoping Reviews
SARS-CoV-2: Severe acute respiratory syndrome coronavirus 2
SCIELO: Online Scientific Electronic Library
SPSS: Statistical Package for the Social Sciences
