# Supplementary material for: Obesity and clinical severity in patients with COVID-19: a scoping review protocol
Source: Syst Rev. 2021 Feb 7;10:51. doi: 10.1186/s13643-021-01603-x (PMC7868111; doi:10.1186/s13643-021-01603-x)
Supplement: Supplementary file 2 — Additional file 2:. Data extraction form [file 13643_2021_1603_MOESM2_ESM.docx]

**Supplementary file 2: Data extraction form**

Reviewer:

Date of data extraction:

**Data to be extracted Item Notes to reviewer**

Publication details

- Authors

Year

Article title

Journal, volume, issue, page numbers

Study design and details

- Setting (country; hospital stay; admission in ICU)
- Sampling technique (convenience sampling; probability sampling)
- Sample size
- Details of study participants (age, sex, disease condition.)
- Design (Controlled Intervention Studies, Observational Cohort and Cross-Sectional Studies, Case-Control Studies and Case Series Studies)
- Method of data collection (interviews; observation; medical record collection)
- Method of data analysis

Specific details of interest for the scoping review

- Diagnostic exam for COVID-19 (RT-PCR or others types of exams)
- Obesity diagnosis (BMI ≥ 30kg/m²)
- Comorbidities
- Reported information of respiratory rate, oxygen saturation or PaO2/FiO2 ratio, information of lung infiltrates
- Presence of severe acute respiratory syndrome (yes or no).
- Reported need of invasive mechanical ventilation (yes or no) and percentage data of need for intubation.
- Reported rates of mortality.

Others outcomes extract from study

- Clarity of interpretation of results
- Discussion of limitations
- Recommendations for future research
- Assessment of generalizability of findings

Theories – only for editorials and expert opinion articles

- Theme addressed in the article
- Theory or possible justification for the increase in clinical severity in obese patients diagnosed with COVID-19
- Literature supporting the authors' theory or justification
- Scientific gaps and suggested future research
- Potential bias, strengths and limitations of included studies.
